# bFGF could be a biomarker of malignancy in RS_3_PE syndrome: an ambispective single-center cohort analysis of 51 patients

**DOI:** 10.1186/s13075-021-02638-0

**Published:** 2021-10-15

**Authors:** Yuzhou Gan, Yi Sun, Jiayang Jin, Yifan Wang, Jiali Chen, Yukchiu Chung, Xue Li, Hua Ye

**Affiliations:** 1 Department of Rheumatology & Immunology and Beijing Key Laboratory for Rheumatism and Immune Diagnosis (BZ0135), Peking University People’s Hospital, 11 Xizhimen South Street, Beijing, 100044 China; 2 Center of Clinical Immunology, Peking University, Beijing, 100044 China; 3 Department of Clinical Laboratory, Peking University People’s Hospital, Beijing, 100044 China

**Keywords:** RS_3_PE, Malignancy, bFGF

## Abstract

**Objectives:**

Remitting seronegative symmetrical synovitis with pitting edema (RS_3_PE) is a rare inflammatory arthritis, with a higher incidence of malignancy. The aim of this study is to identify biomarkers for predicting malignancy in RS_3_PE.

**Methods:**

A total of 51 patients with RS_3_PE from September 2007 to May 2019 were retrospectively reviewed and followed for up to 5 years, with 15 patients with osteoarthritis (OA) and 14 patients with elderly-onset rheumatoid arthritis (EORA) as disease controls. Serum levels of angiogenesis cytokines were measured by electrochemiluminescent immunoassay and Luminex Human Magnetic Assay. Clinical data and laboratory parameters were analyzed to identify risk factors for malignancy.

**Results:**

A total of forty-eight RS_3_PE patients (94.1%) were available with follow-up data; 8 patients (16.7%) were diagnosed with malignancy, of which 6 patients were hematological tumor; and 2 patients were solid tumors. Serum levels of basic fibroblast growth factor (bFGF) were exclusively higher in RS_3_PE patients with malignancy [14.21 (7.52, 23.18) ng/mL] than RS_3_PE patients without malignancy [4.32 (2.88, 7.42) ng/mL], OA [3.20 (2.20, 5.30) ng/mL], and EORA [3.20 (2.20, 5.30) ng/mL]. The optimal cut-off value of bFGF for malignancy was 10ng/mL in RS_3_PE. Logistic regression analysis indicated that elevation of bFGF was a risk factor for malignancy in RS_3_PE.

**Conclusions:**

This study indicated that bFGF was elevated in RS_3_PE patients with malignancy and could serve as a biomarker for predicting paraneoplastic RS_3_PE.

## Introduction

Remitting seronegative symmetrical synovitis with pitting edema (RS_3_PE) is a rare elderly-onset inflammatory arthritis, characterized by symmetrical involvement of small joints and marked pitting edema on the dorsum of the hands and feet [1, 2]. In addition, a higher incidence of malignancy was reported in RS_3_PE after the first symptom onset or during follow-up [2–4]. However, no significant demographic or clinical differences were observed between idiopathic and paraneoplastic cases of RS_3_PE, which suggests the importance of investigating novel serum tumor markers.

Previous studies have found two angiogenesis cytokines, namely vascular endothelial growth factor (VEGF) and matrix metalloproteinase (MMP)-3, were involved in the pathogenesis of RS_3_PE along with malignancy [5–8]. But later findings indicated that elevated levels of VEGF were also characteristics of infections and organizing pneumonia in RS_3_PE [9, 10]. MMP-3 was elevated in RS_3_PE patients with solid malignancy [11], but it was also an indicator for active arthritis [12]. It still remains elusive whether there is a specific biomarker for identifying arthritis with malignancy.

Since angiogenesis plays an important role in the pathogenesis and progression of cancer, we simultaneously evaluated the serum levels of 12 angiogenesis cytokines in paraneoplastic RS_3_PE, comparing with idiopathic RS_3_PE, osteoarthritis (OA), and elderly-onset rheumatoid arthritis (EORA) in the current study. We aimed to discover some novel markers for predicting malignancy in RS_3_PE.

## Methods

### Patients enrolled

A single-center cohort study was performed in the Department of Rheumatology, Peking University People’s Hospital. Fifty-one patients diagnosed with RS_3_PE syndrome were consecutively enrolled from September 2007 to May 2019, fulfilling the following criteria: (1) bilateral pitting edema of dorsum of hands and/or feet, (2) abrupt onset of polyarthritis, (3) age > 50 years, and (4) seronegative for rheumatoid factor (RF) [13]. As disease controls, 15 patients with OA and 14 patients with EORA were also enrolled, with sex- and age-matched. All the participants in disease control groups were excluded from malignancy.

### Study design and data collection

Patients with RS_3_PE were followed up for 5 years or monitored up to February 29, 2021, if they enrolled after February 28, 2016. The primary clinical outcome was the occurrence of malignancy. The baseline clinical and laboratory characteristics, coexistence of malignancy, and response to treatment were obtained from the medical records. If no follow-up data was available in our center, we contacted the family members to acquire the physical status (especially the occurrence of malignancy) confirmed by regular medical examination reports.

### Measurement of angiogenesis cytokines

The serum samples were collected from 45 RS_3_PE patients and all disease controls at baseline and stored at −80°C in polypropylene microfuge tubes without thawing before the test. Serum levels of VEGF-A, VEGF-C, VEGF-D, FMS-like tyrosine kinase 1 (Flt-1), Tie-2, placental growth factor (PIGF), and basic fibroblast growth factor (bFGF) were measured via electrochemiluminescent immunoassay by V-PLEX Plus Angiogenesis Panel 1 Human Kit (Meso Scale Discovery). Serum levels of MMP-1, MMP-3, MMP-7, mesothelin, and tumor necrosis factor-related apoptosis-inducing ligand (TRAIL) were determined with Luminex Human Magnetic Assay (5-Plex) LXSAHM-05 (R&D).

### Statistical analysis

Data analyses were performed using SPSS 23.0 for Windows. Continuous data with the normal distribution were expressed as the mean ± standard, and differences between groups were analyzed by one-way ANOVA. Continuous data with skewed distribution were expressed as median (P25, P75), and differences between groups were analyzed by Kruskal-Wallis test. Dichotomous variables were reported as frequency (percentages), and differences between groups were compared using the chi-square test (or Fisher’s exact test when appropriate). The cut-off value of bFGF in RS_3_PE patients with malignancy was determined by receiver operating characteristic (ROC) methods. Univariate and multivariate logistic regression analyses were adopted to identify risk factors of malignancy. The variables assessed in the univariate regression analysis were entered as independent variables in multivariate logistic regression analysis when *P* value <0.1. Two-sided *P* < 0.05 was considered statistically significant. The *P* value was adjusted by Bonferroni correction in multiple tests.

## Results

### Clinical and laboratory features of RS_3_PE patients

The clinical and laboratory features of overall RS_3_PE patients were shown in Table 1. Forty-eight patients (94.1%) were available with follow-up data, and a total of 26 patients (54.1%) completed a 5-year follow-up, and the follow-up time for the remaining 22 patients ranged from 1 month to 54 months. During the study period, eight of them (16.7%) were diagnosed with malignant tumors. Twenty-eight patients (54%) were male, and the average age at onset was 73.24±9.23 years. Pitting edema was seen in the hands of 45 patients (88.2%) and in the feet of 27 patients (52.9%). Weight loss was seen in 19 patients (37.3%). Patients had an elevated level of C-reactive protein (CRP) (43.9 [22.8, 82.0] mg/dL) and erythrocyte sedimentation rate (ESR) (55.37±34.12 mm/h). All patients had a normal level of carcinoembryonic antigen (<4.7 ng/mL). Eight patients (17.4%) had an elevated level of neuroenolase (>16.3ng/mL), 3 patients (5.8%) had an elevated level of cytokeratin 19 fragment (>3.3ng/mL), 2 patients (3.9%) had an elevated level of carbohydrate antigen 19-9 (>39U/mL), and only one patient (1.9%) had an elevated level of alpha-fetoprotein (>7ng/mL). Antinuclear antibody (ANA) was positive (≥1:80) in 6/51 (11.8%), and anti-Ro-52 was positive in 5/51 (9.8%). The median of the initial prednisolone dose was 15 mg/day, and 44/51 (89.8%) showed a good response to prednisolone.

**Table 1:** Characteristics of RS_3_PE patients with or without malignancy

	Overall (*n* = 51)	RS_3_PE with malignancy (*n* = 8)	RS_3_PE without malignancy (*n* = 40)	*P*
Age (years)	73.39±9.18	75.00±11.07	73.50±8.51	0.667
Age of onset (years)	73.24±9.23	74.5±11.30	73.4±8.53	0.754
Gender (M/F)	28/23	5/3	21/19	0.710
Pitting edema of hands (*n*, %)	45 (88.2)	7 (87.5)	35 (87.5)	1
Bilateral (*n*, %)	42 (93.3)	5 (71.4)	34 (97.1)	0.067
Weight loss (*n*, %)	18 (35.3)	5 (62.5)	12 (30)	0.112
CEA > 4.7 ng/mL	0^a^	/	/	/
AFP > 7ng/mL	1 (2.6)^a^	1 (14.3)^c^	0^b^	0.152
CA19-9 > 39U/mL	2 (5.1)^a^	0^c^	2 (5.1)^b^	1
CYFRA21-1 >3.3 ng/mL	3 (6.5)^a^	1 (14.3)^c^	2 (5.1)^b^	0.398
NSE >16.3 ng/mL	8 (17.4)^a^	2 (28.6)^c^	6 (18.2)^b^	0.587
C reactive protein (mg/L)	43.9 (22.8, 82.0)	35.9 (12.0, 91.0)	45.7 (26.4, 84.9)	0.674
Erythrocyte sedimentation rate (mm/h)	55.37±34.12	48.88±29.00	56.85±35.81	0.558
Immunoglobulin A (g/L)	3.05 (1.62, 3.73)	2.71 (1.18, 3.66)	3.13 (1.62, 4.04)	0.734
Immunoglobulin G (g/L)	12.42±4.74	12.56±5.24	12.32±4.81	0.900
Immunoglobulin M (g/L)	0.71 (0.50, 1.09)	0.68 (0.50, 1.39)	0.71 (0.47, 1.12)	0.968
Complement 3 (g/L)	1.04 (0.87, 1.28)	1.03 (0.79, 1.16)	1.05 (0.88, 1.29)	0.422
Complement 4 (g/L)	0.23±0.09	0.25±0.04	0.24±0/09	0.722
ANA≥1:80 (*n*, %)	6 (11.8)	0	6 (15)	0.571
Anti-Ro-52 (*n*, %)	5 (9.8)	1 (12.5)	4 (10)	1
Initial prednisolone dose	15 (15, 30)	15 (10, 30)	15 (15, 27.5)	0.946
Fast response to prednisolone (*n*, %)	44 (89.8)	3 (42.8)^d^	38 (97.4)	0.001^*^

Values displayed as *n* (%), mean ±standard deviation, or median (P25, P75) according to their features of the distribution

*Note*: *RS*_*3*_*PE* remitting seronegative symmetrical synovitis with pitting edema, *ANA* anti-nuclear antibody, *CEA* carcinoembryonic antigen, *AFP* alpha-fetoprotein, *CA19-9* carbohydrate antigen 19-9, *CYFRA21-1* cytokeratin 19 fragment, *NSE* neuroenolase

^*^P<0.05, a significant difference between RS_3_PE patients with and without malignancy

^a^Five patients did not have the data of serum tumor markers

^b^Two patients did not have the data of serum tumor markers

^c^One patient did not have the data of serum tumor markers

^d^One patient did not receive glucocorticoids

### Comparison between RS_3_PE patients with and without malignancy

The detailed clinical profiles of the eight RS_3_PE patients with malignancy were displayed in Table 2. The prevalence of malignancy was 16.7% (8/48), six were hematological tumors, and 2 were solid tumors. The time from the onset of arthritis to confirmation of malignancy was from 2 months to 3 years. In these 6 patients with hematological tumors, 4 patients were diagnosed within 6 months from arthritis onset, and 3 patients showed poor response to low-dose prednisolone. Both patients with solid tumors were diagnosed 2 years after arthritis onset, and one of them (50%) was resistant to low-dose prednisolone.

**Table 2:** Detailed clinical profiles of RS_3_PE patients with malignancy

No.	Age/sex	Type of malignancy	Time from arthritis onset to malignancy confirmation	Signs	ESR (mm/h)	CRP (mg/L)	bFGF (ng/mL)	Initial pred. (mg/day)	Response to pred.
1	85/M	Acute myeloid leukemia -M2	6 months	Unilateral hands	29	16.9	25.04	0	/
2	75/F	Multiple myeloma (IgA, *λ*)	2 years	Bilateral hands	61.2	70	24.35	15	poor
3	80/M	Diffuse large B cell lymphoma	2 months	Bilateral hands	86	45.9	10.2	40	poor
4	53/M	Plasma cell leukemia	8 months	Unilateral hands	19	177.3	42.48	30	poor
5	85/F	Rectal carcinoma	3 years	No hands	44	10.4	17.58	30	poor
6	66/F	Multiple myeloma	3months	Bilateral hands	71	26.11	10.94	15	good
7	83/M	Non-Hodgkin lymphoma	2 months	Bilateral hands	67	101	5.57	15	good
8	73/M	Lung carcinoma	2 years	bilateral hands	5	9.39	6.63	7.5	good

*RS*_*3*_*PE* remitting seronegative symmetrical synovitis with pitting edema, *CRP* C-reactive protein, *ESR* erythrocyte sedimentation rate, *Pred*. prednisolone

We next compared the clinical and laboratory features between patients with or without malignancy (Table 1). Better response to prednisolone was found in patients without malignancy (*n*=38/40, 97.4%) than patients with malignancy (*n*=3/7, 42.8%). However, significant differences were not seen in demographic figures (age and gender), clinical features (patterns of edema and weight loss), and laboratory features (CRP, ESR, immunoglobulin, complement, and tumor markers).

### Serum levels of angiogenesis cytokines among RS_3_PE with/without malignancy, OA, and EORA

**Fig. 1:**
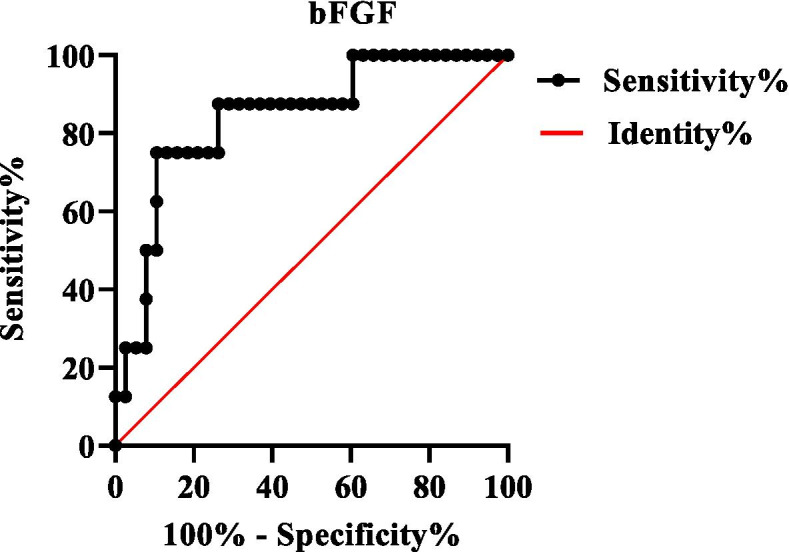
ROC curve for bFGF in predicting malignancy in patients with RS_3_PE. ROC receiver operating characteristic, *bFGF* basic fibroblast growth factor, *RS*_*3*_*PE* remitting seronegative symmetrical synovitis with pitting edema

Twelve angiogenesis cytokines were measured, and the results were demonstrated in Table 3. Serum levels of bFGF were exclusively higher in RS_3_PE patients with malignancy [14.21 (7.52, 23.18) ng/mL] than RS_3_PE patients without malignancy [4.32 (2.88, 7.42) ng/mL], OA [3.23 (1.96, 5.59) ng/mL], and EORA [3.20 (2.20, 5.30) ng/mL]. However, there were no significant differences in serum levels of VEGF-A, VEGF-C, VEGF-D, Flt-1, Tie-2, PIGF, MMP-1, MMP-3, MMP-7, mesothelin, and TRAIL among different groups. Figure 1 showed the ROC curve of bFGF with an AUC value of 0.817, and the optimal cut-off value was 10ng/mL; the sensitivity was 75% and the specificity was 89.5%.

**Table 3:** Comparison of angiogenesis cytokines among RS_3_PE with/without malignancy, OA, and EORA

	RS_3_PE with malignancy (*n* = 8)	RS_3_PE without malignancy (*n* = 38)	OA (*n* = 15)	EORA (*n* = 14)	F/t	*P*
Flt-1 (ng/mL)	7.38±2.64	6.14±2.25	5.96±1.53	5.59±1.67	1.321	0.275
PlGF (ng/mL)	6.14 (4.436, 6.58)	5.99 (4.45, 7.29)	5.61(4.74, 6.6)	4.81 (4.48, 7.15)	0.745	0.863
Tie-2 (ng/mL)	8.99±2.34	9.92±2.89	11.26±2.72	8.47±2.41	**2.816**	**0.045**
VEGF (ng/mL)	168.41 (90.86, 327.61)	83.15 (50.34, 200.11)	156.24 (117.94, 239.03)	71.68 (40.74, 234.95)	4.144	0.246
VEGF-C (ng/mL)	3.76 (1.69, 7.58)	4.75 (3.72, 9.79)	6.57 (5.45, 7.82)	5.31 (3.87, 6.46)	3.281	0.350
VEGF-D (ng/mL)	18.40 (14.86, 30.31)	31.92 (26.88, 46.75)	26.43 (23.64, 36.74)	24.98 (17.56, 34.74)	**10.433**	**0.015**
bFGF (ng/mL)	14.21 (7.52, 23.18)	4.32 (2.88, 7.42)^▲^	3.23 (1.96, 5.59)^▲^	3.20 (2.20, 5.30)^▲^	**15.861**	**0.001**
MMP-3 (ng/mL)	24.74 (17.96, 51.67)	36.08 (19.01, 54.34)	22.03 (14.84, 25.57)	33.31 (15.86, 41.44)	5.346	0.148
MMP-1 (ng/mL)	4.98 (3.80, 7.50)	3.93 (1.94, 7.73)	2.90 (1.72, 4.50)	5.80 (3.37, 10.73)	5.472	0.140
MMP-7 (ng/mL)	2.11 (1.55, 5.02)	2.86 (1.99, 3.40)	2.68 (1.64, 4.16)	3.06 (2.02, 5.03)	0.864	0.834
TRAIL (pg/mL)	82.28 (57.26, 121.38)	93.86 (75.98, 140.34)	98.56 (84.51, 114.28)	89.96 (61.04, 124.94)	2.324	0.508
Mesothelin (ng/mL)	25.65 (17.81, 34.84)	24.10 (17.89, 30.54)	27.20 (15.40, 30.27)	21.96 (18.94, 28.50)	0.688	0.876

*Note*: ^▲^Significance comparing with RS_3_PEpatients with malignancy, adjusted *P*<0.05. *RS*_*3*_*PE* remitting seronegative symmetrical synovitis with pitting edema, *Flt-1* Fms-like tyrosine kinase 1, *VEGF* vascular endothelial growth factor, *PIGF* placental growth factor, *bFGF* basic fibroblast growth factor, *MMP* matrix metalloproteinase, *TRAIL* tumor necrosis factor-related apoptosis-inducing ligand

### Risk factors for malignancy in RS_3_PE

As shown in Table 4, the results of univariate logistics models found that bilateral pitting edema of the hands (OR=0.074, 95%CI (0.006–0.968), *P*=0.047) and good response to prednisolone (OR=0.039, 95%CI (0.005–0.311), *P*=0.002) were negatively associated with malignancy in RS_3_PE, and elevation of bFGF (>10ng/ml) (OR=14.084, 95%CI (2.421–83.332), *P*=0.003) was positively associated with malignancy in RS_3_PE. Then, the multivariate logistic models showed elevation of bFGF was a unique risk factor for malignancy (OR=14.667, 95%CI (2.029–106.038), *P*=0.008).

**Table 4:** Risk factors for malignancy in RS3PE by logistic models

Variables	Univariate analysis			Multivariate analysis		
	*B*	OR (95%CI)	*P*	*B*	OR (95%CI)	*P*
Male	0.411	1.508 (0.317–7.177)	0.606			
Bilateral pitting edema of hands	−2.61	0.074 (0.006–0.968)	**0.047**	−1.255	0.285 (0.035–2.320)	0.068
Good response to prednisolone	−3.232	0.039 (0.005–0.311)	**0.002**	−1.967	0.140 (0.017–1.182)	0.071
bFGF>10ng/ml	2.651	14.084 (2.421–83.332)	**0.003**	2.686	14.667 (2.029–106.038)	**0.008**

*RS*_*3*_*PE* remitting seronegative symmetrical synovitis with pitting edema, *bFGF* basic fibroblast growth factor

## Discussion

In the present study, we reviewed the clinical and laboratory features and simultaneously analyzed multiple angiogenesis cytokines in RS_3_PE patients with malignancy. We found elevation of bFGF might be a useful predictor for malignancy in RS_3_PE.

Increased associated malignancy in RS_3_PE has been reported since 1985, including hematological malignancies and solid tumors [2, 14–16], and the average malignancy rate was estimated to 20% [2], which is similar to our study. Although hematological malignancies were the primary tumors in our study and most of them were diagnosed within the first 6 months, both two associated solid tumors were confirmed during the follow-up. Besides, a French study of six men with RS_3_PE demonstrated that all solid malignancy was discovered during a 5-year follow-up [17]. These findings indicate that solid tumors might be relatively insidious in RS_3_PE-related malignancies, reminding rheumatologists the importance of tumor screening during the follow-up.

Poor response to low-dose prednisolone is associated with malignancies in RS_3_PE in our study, and some reported cases of paraneoplastic RS_3_PE are also revealed poor response to glucocorticoid [2, 18]. However, rapid response to glucocorticoid therapy is also found for some paraneoplastic RS_3_PE, and there are no clinical variables for predicting malignancy in RS_3_PE [1, 3, 4, 19], which calls for more effective biomarkers.

Interestingly, our study discovered bFGF is the only angiogenesis cytokine which is elevated particularly in RS_3_PE-associated malignancy, and further multiple logistic regressions revealed elevation of bFGF may serve as a marker for predicting malignancy in RS_3_PE. bFGF, also known as fibroblast growth factor 2 (FGF-2), is one of the prototypes of the FGF family, which signals through FGF receptors (FGFRs) and promotes growth and differentiation of a broad spectrum of cell types, including dermal fibroblasts, keratinocytes, endothelial cells, and melanocytes [20–22]. In addition, bFGF also plays a critical role in promoting tumor angiogenesis and metastasis and has been shown to be involved in the invasion and progression of solid and hematological malignancies [21, 23–26].

Apart from tumor genesis, it has also been found that bFGF could stimulate osteoclastogenesis and promote bone absorption through binding to FGFRs, and is the only one of the bone-resorptive cytokines that are highly expressed in the synovial fluid of RA patients [27–29]. Thus, significantly higher serum bFGF in RS_3_PE may reflect the secretion of bFGF in situ of tumor tissues as well as synovium, suggesting that bFGF might play an important role in the pathogenesis of RS_3_PE. Besides, the titers of bFGF were relatively lower in the RS_3_PE-associated solid malignancy (confirmed after 2 years from arthritis onset) than RS_3_PE-associated hematological malignancy (confirmed within 1 year from arthritis onset), which might partially due to the late onset of solid tumor.

Previous researches have pointed out that RS_3_PE might be a VEGF-associated disorder and elevated serum level of VEGF was also found in paraneoplastic RS_3_PE [6, 7]. However, a recent study has found serum VEGF is elevated in many elderly patients with different rheumatic diseases, indicating that VEGF may not be a marker for predicting malignancy [30]. Tomoki et al. reported high-serum MMP-3 is a characteristic of RS_3_PE patients with neoplasm. Nevertheless, serum levels of MMP-3 are relatively lower in our paraneoplastic RS_3_PE patients compared with non-paraneoplastic RS_3_PE patients. This difference might be partially due to different kinds of malignancy. All of the cancers in Tomoki et al.’s study are solid tumors, and they merely compared the difference of serum MMP-3 between patients with and without malignancy [11]. Most of our paraneoplastic RS_3_PE patents are hematological, and we utilized multiple logistic models to fully confirm the relationship between bFGF and malignancy.

### Limitations

Due to the rarity of RS_3_PE, the number of associated malignant cases is relatively less at a single center; therefore, a prospective cohort or multi-center studies are needed to confirm the clinical significance of bFGF in further studies.

## Conclusion

Our study revealed the clinical significance of serum bFGF in RS_3_PE; thus, bFGF might be associated with malignancy in RS_3_PE. Further research might verify our findings by multi-center studies and explore the prognostic value for angiogenesis cytokines.

## Data Availability

Dr. Hua Ye and Dr. Yuzhou Gan had full access to all of the data in the study and take responsibility for the integrity of the data and the accuracy of the data analysis

## Abbreviations

bFGF: Basic fibroblast growth factor
EORA: Elderly-onset rheumatoid arthritis
Flt-1: FMS-like tyrosine kinase 1
MMP: Matrix metalloproteinase
OA: Osteoarthritis
PIGF: Placental growth factor
RF: Rheumatoid factor
ROC: Receiver operating characteristic
RS_3_PE: Remitting seronegative symmetrical synovitis with pitting edema
TRAIL: Tumor necrosis factor-related apoptosis-inducing ligand
VEGF: Vascular endothelial growth factor
